# Health-related quality of life of children and adolescents with the most common ectodermal dysplasia: focus group study and item development for a condition-specific patient-reported outcome measure

**DOI:** 10.1186/s13023-026-04246-0

**Published:** 2026-02-06

**Authors:** Cosima Kügler, Stefanie Witt, Johanna Hammersen, Julia Quitmann, Holm Schneider

**Affiliations:** 1 Center for Ectodermal Dysplasias, University Hospital Erlangen, Loschgestr. 15, 91054 Erlangen, Germany; 2 Department of Pediatrics, Friedrich-Alexander-Universität Erlangen-Nürnberg, Erlangen, Germany; 3 Center for Psychosocial Medicine, Department of Medical Psychology, University Medical Center Hamburg-Eppendorf, Hamburg, Germany; 4 Department of Social Work, Faculty of Business and Social Sciences, University of Applied Sciences Hamburg, Hamburg, Germany

**Keywords:** Health-related quality of life, Hypohidrotic ectodermal dysplasia, Patient-reported outcome measure, Questionnaire

## Abstract

**Background:**

Hypohidrotic ectodermal dysplasia (HED) includes some rare congenital disorders affecting the skin, its appendages, and the teeth. Although hypohidrosis can be life-threatening, research on the impact of HED on the patient’s quality of life has been very limited so far. Aiming at the development of a condition-specific patient-reported outcome measure (PROM) assessing health-related quality of life (HRQoL), we studied the HRQoL of children and adolescents with HED.

**Methods:**

Focus (group) interviews were conducted with patients at the age of 8 to 17 years and parents of patients aged 2–17 years, all recruited from the HED patient registry of the University Hospital Erlangen, Germany. A qualitative interview analysis was performed, identifying key themes and generating a category system based on relevant interview excerpts. Using the Card-sorting method, an item list for the pilot version of the questionnaire was made.

**Results:**

Eleven focus (group) interviews with 9 children/adolescents and 22 parents provided information on 24 patients. The analysis identified 562 statements about HRQoL, which were categorized into six main domains: physical well-being, emotional well-being, social well-being, autonomy, childcare/school/education, and parental well-being. On the basis of these statements, age-adjusted pilot versions of a questionnaire were developed, consisting of 83 items each: (1) an observer report for parents of children aged 2–7 years, (2) a self-report combined with an observer report for children and adolescents aged 8–17 years.

**Conclusions:**

This study is the first to explore HRQoL of children and adolescents with HED through qualitative interviews. Our findings highlight the impact of heat intolerance on daily life, the emotional burden of physical limitations, and the crucial role of coping strategies, social inclusion, and supportive relationships. The final validation of the new PROM, which shall enable the systematic integration of patient perspectives into clinical practice and research, is underway.

## Abstract (significance)

What’s already known about this topic?

What does this study add?

Previous research has demonstrated that hypohidrosis and lack of teeth significantly impair daily life and HRQoL in individuals with HED. In addition, psychosocial burdens, such as stigmatization and social withdrawal resulting from phenotypic differences, have been identified as relevant factors.

This study provides deeper insights into the HRQoL of children and adolescents with HED based on qualitative interviews with both affected individuals and their parents, which captured a broad range of subjective experiences and unmet needs. It led to the development of a condition-specific PROM that should enable a more systematic consideration of these perspectives both in clinical practice and research.

## Introduction

Ectodermal dysplasias (ED) are a heterogeneous group of genetic disorders characterized by the abnormal development of at least two structures derived from the embryonic ectoderm, such as skin, hair, nails, teeth, and certain glands [1]. The minimum birth prevalence of ED was recently estimated to be 14.5 per 100 000 live births [2].

The most common form of ED is X-linked hypohidrotic ED (XLHED), also known as Christ-Siemens-Touraine syndrome. Clinically relevant symptoms of this disorder include heat intolerance and hyperthermic episodes, chewing and speaking problems due to missing or malformed teeth, frequent nasal and ear obstructions, sicca symptoms (dry mouth and dry eyes), and dry, frequently inflamed skin. Patients typically exhibit thin scalp hair, sparse or absent eyebrows and eyelashes, and distinct facial features, e.g., a prominent forehead, saddle nose, and everted lips. As the disorder is X-linked, male subjects are usually more severely affected than females [2]. The symptoms of XLHED can be associated with a significantly reduced quality of life of affected individuals. In the medical context, quality of life typically refers to the concept of health-related quality of life (HRQoL), defined as a multidimensional construct that pertains to well-being and functional ability from the perspective of patients and/or observers. Fundamental dimensions include physical, emotional, mental, social, and daily functional components [3]. HRQoL comprises factors, such as disease symptoms, and the subjective evaluation of these factors [4].

Patient-reported outcome measures (PROMs) of HRQoL are broadly classified as either generic or condition-specific [5]. Generic PROMs assess HRQoL across a wide range of health conditions, thereby enabling comparisons between healthy individuals and patients with such health conditions or among patient groups with different diseases. In contrast, condition-specific instruments measure HRQoL in the context of a specific disease [5]. The latter approach offers the opportunity to depict the impact of a health condition on the lives of affected individuals more specifically and to detect small but meaningful changes in disease symptoms and the associated subjective limitations more sensitively [5].

The systematic integration of PROMs assessing HRQoL into routine care offers an important tool for enhancing patient-centered and value-based care. Benefits include, but are not limited to, improved patient-physician communication, more sensitive monitoring of disease progression, and personalization of both medical therapy and mental health support [6]. In the context of rare diseases, the routine assessment of PROM data provides a structured framework for identifying unmet patient needs, facilitating shared decision-making, and detecting subtle changes in a patient’s status that may be absent from traditional clinical metrics. Consequently, PROM data contribute to a more comprehensive and holistic understanding of the patient’s lived experience, which may allow improvement of both health outcomes and care quality [3, 4]. Especially in the case of developmental disorders with symptoms that are not always obvious and cannot be measured easily, assessing disease-specific dimensions of HRQoL can help to address the clinical challenges.

ED represents a group of disorders with diverse and even life-threatening symptoms that can severely restrict daily life from early infancy to adulthood [7]. Therefore, the aspects mentioned above are of great importance. During conversations between doctor and patient or doctor and families, there is a risk that individually relevant aspects may be overlooked and symptoms may be misjudged in terms of their impact on patients’ HRQoL. However, research on the impact of ED on the HRQoL of children and adolescents has been very limited so far. Some publications indicate that reduced or absent sweating affects the daily life and HRQoL of individuals with hypohidrotic ED (HED) [7–10]. One study examined psychosocial aspects, showing that mockery and avoidance by peers, primarily due to different appearances, do occur [11]. Most studies on the quality of life of patients with ED, however, focus on oral HRQoL [12–14] and neglect other important aspects of HRQoL, such as effects on autonomy, school life and overall life satisfaction.

The development of a disease-specific diagnostic instrument is of particular importance in the context of rare diseases like HED. Research into rare conditions frequently faces two fundamental challenges: significant symptom heterogeneity and a pronounced lack of validated, tailored outcome measures [15]. Generic HRQoL PROMs often do not adequately capture the unique and multifaceted disease burden experienced by patients with a rare disorder, as such burdens are often specific to the condition’s pathophysiology and its social and functional implications. This discrepancy in measurement hinders the progress of clinical research, because it impedes the ability to detect treatment effects, and it also limits the comprehensive assessment of patients in clinical practice. The objective of our study was to develop a (pilot) questionnaire as the first step towards a condition-specific PROM that reflects the specific experiences of individuals with HED for both self-report and parent report, based on the FDA guideline for PROM development [16]. This will address the aforementioned gap directly. It may not only contribute to improved care for patients with HED but also serve as a model for outcome measure development in other rare diseases, where patient-centered research is paramount yet methodologically challenging.

## Materials and methods

### Project phases

The Expert Group for Ectodermal Dysplasias including incontinentia pigmenti and p63-associated disorders of the European Reference Network (ERN) Skin initiated the development of an ED-specific HRQoL questionnaire for children and adolescents in 2018. The first conceptual framework phase comprised an open survey of parents and a survey of experts: In the protected online forum of the German-Austrian ED patient organization (Selbsthilfegruppe Ektodermale Dysplasie e.V.), all parents were asked to list the aspects that they considered relevant for their children in response to the question “In which areas does your children’s ED affect their life?” The collected responses were then presented by the chairperson of the patient organization to an expert panel of two pediatricians and two dermatologists, all of whom had many years of experience in caring for children and adolescents with ED. The panel asked the two pediatricians to draft a preliminary, non-validated questionnaire containing 20 questions.

In the summer of 2019, the first preliminary questionnaire was tested for comprehensibility and relevance to the patient community: It was sent to 68 parents with the request that their affected children/adolescents complete it themselves or, in the case of younger children, complete it together with the parents, noting any content that was unclear or considered unimportant. The medical experts then evaluated the returned questionnaires resulting in a more comprehensive questionnaire version with 78 questions (2–7 years) and 89 questions (8–18 years) (second preliminary questionnaire). In 2021, a collaboration between the Center for Ectodermal Dysplasias at the University Hospital Erlangen (ZEDER), Germany, led by Prof. Dr. Holm Schneider, and the Quality of Life Working Group at the Department of Medical Psychology of the University Medical Center Hamburg-Eppendorf, Germany, led by Prof. Dr. Julia Quitmann, was established to address the previously existing lack of expertise in questionnaire development and to develop and validate a final ED-specific questionnaire (ED-QoL) as a PROM to assess HrQoL in pediatric patients with ED according to the FDA guidance for PROM development [16]. Since the first conceptual framework phase only included a parent survey, we planned a second phase based on focus (group) interviews also with the affected children to capture their perspective, a key criterion for PROM development [16].

This second phase involved a preliminary scoping review to identify relevant HRQoL domains and potential items for the target population. The scoping activity, which informed the foundational framework for our study, was conducted as part of an unpublished academic thesis project. While the full results of that thesis are not publicly cited, its findings, specifically, the synthesis of key publications and the preliminary identification of relevant HRQoL domains, directly informed the subsequent stages of ED-QoL development.

Based on the findings of the focus (group) interviews and the preliminary scoping review, a new item list was developed independently of the preliminary questionnaire. Although this initial questionnaire was not retained in its original form, it served as the basis for a cognitive debriefing, during which we were able to gather feedback on, for instance, the clarity of the wording, the appropriateness of the response options, and the suitability of the recall period. Such feedback influenced the design of a more mature questionnaire. A pilot test with cognitive debriefing and a field study and re-test for the final psychometric validation of the PROM is currently being conducted [16]. This paper reports the results of the second conceptual framework phase and item generation based on qualitative focus (group) interviews with ED patients aged 8–17 years and parents of patients between 2 and 17 years of age.

### Patient recruitment

Potential participants in the qualitative focus (group) interviews were recruited via convenience sampling through the HED patient registry of the University Hospital Erlangen. This method may have favored the participation of individuals and families who are highly engaged with support networks and may not fully capture the experiences of those less connected to the patient community. Inclusion criteria were: (1) a molecular genetic diagnosis of a hypohidrotic ED, (2) pediatric ED patients older than 7 years (self-report) or parents of patients between the age of 2 and 17 years (parent report), and (3) sufficient German language skills to participate in an interview. Sufficient knowledge of German language was defined as the ability to understand complex information and engage in a fluent conversation about emotionally charged topics without needing an interpreter. Due to the nature of the patient registry, which primarily lists subjects affected by XLHED, all interviewees had been diagnosed with XLHED. After initial telephone contact, detailed written participant information was provided, and written consent was obtained. Participants were stratified by age (2–7 years, 8–12 years, and 13–17 years), biological sex (male, female), and self-report vs. observer report. In the group of children between 2 and 7 years of age, only parents were interviewed. Children or adolescents of the other two groups and their parents participated in separate interviews. Interviews were conducted either individually or in focus groups, depending on the participant’s preference and logistical feasibility. Each participant could choose between the two options and was assured of confidentiality before the interview began. Collected data included sociodemographic information of the patients (age, sex, occurrence of ED in the family) and clinical data related to the condition (medical history, severity of symptoms).

### Data collection

The focus (group) interviews were conducted as video conferences (using Jitsi Meet) and followed a semi-structured interview guideline. The primary method employed was open-ended elicitation, which enabled participants to freely express and prioritize the dimensions of HRQoL most relevant to them, thereby safeguarding against the imposition of researcher preconceptions. Furthermore, to systematically capture all patient-important aspects, the interview concluded with a non-leading prompt for any additional topics, followed by targeted yet separate probes for the domains of eating and sleeping, if these topics had not been raised spontaneously. All interviews were conducted by a trained interviewer who had received comprehensive training based on a study-specific interviewer manual. The training was supervised by an experienced senior qualitative researcher. The interviewer underwent a thorough instruction in moderation techniques for semi-structured interviews, with the focus on asking open-ended questions, active listening, and maintaining a neutral, non-judgmental stance. The senior researcher supervised the first interview in this study to provide direct feedback. Furthermore, the senior researcher acted as a continuous supervisor throughout the data collection period, serving as a central contact person to discuss challenges and ensure adherence to the protocol. The interviews were divided into two parts, each approximately 45 min long. The first part consisted of structured questions to gather information on the impact of various disease factors of XLHED on HRQoL. It included 20 questions for parents and 24 for children and adolescents. In the second part, the so-called cognitive debriefing, children and parents were asked to discuss the individual questions’ comprehensibility, completeness, and relevance based on a previously added cognitive debriefing part of the second preliminary questionnaire which they had filled out before. The audio track of each focus group interview was digitally recorded using a separate audio recorder.

### Data analysis

All interviews were subjected to a qualitative focused interview analysis [17], where the material was analyzed deductively and inductively based on a pre-developed category system relevant to the research question. The recorded focus (group) interviews were transcribed verbatim and anonymized using the MAXQDA 2022 software [18]. Relevant text passages related to HRQoL were assigned to the deductively formed categories of the main dimensions of HRQoL identified in the preliminary scoping review. Subcategories were developed inductively. Finally, the categories were evaluated and discussed following the methodology of Card sorting [19], leading to the initial item list for the pilot version.

To ensure coding reliability, two researchers independently coded 20% (*n* = 2) of the transcripts. Coding discrepancies were identified through regular comparison of the applied codes and subsequently discussed in alignment meetings until a consensus was reached. In cases where no consensus could be achieved, a third senior researcher was consulted to adjudicate the final code based on the predefined codebook definitions.

All quotations and items in this manuscript were derived from German interviews. They were translated into English by the authors, so the English version does not adhere to the “Principles of Good Practice for the Translation and Cultural Adaptation Process for PROM” [20].

The study as detailed above was approved by the Ethics Committee of the Friedrich-Alexander-Universität Erlangen-Nürnberg (reference number 21-412-B).

## Results

In this study, 38 families were contacted, of which 21 took part in the focus (group) interviews (response rate: 55%). Reasons for non-participation included unavailability or a conscious decision against participating in the interviews. Eight focus group meetings and three focus interviews were conducted from February 16, 2022, to June 23, 2022 (Table 1). The distribution of participants by age group, sex, and self-report vs. observer-report is given in Table 2. Nine children and adolescents and 22 parents were interviewed, providing information on 24 children and adolescents (18 male and six female). Sixteen patients had severe symptoms (anhidrosis or severe hypohidrosis and oligo-/anodontia) (Table 3).

**Table 1:** Characteristics of the focus (group) interviews

Focus group interview		Age group (years)		Parents				Children		
				male		female		male		female
1		2–7		n.a.		3		n.a.		n.a.
2		2–7		2		2		n.a.		n.a.
3		8–12		n.a.		4		n.a.		n.a.
4		8–12		n.a.		n.a.		4		n.a.
5		13–17		1		4		n.a.		n.a.
6		13–17		n.a.		n.a.		2		n.a.
7		8–17		n.a.		4		n.a.		n.a.
8		8–12		n.a.		n.a.		n.a.		2
**Focus interview**										
1		2–7		n.a.		1		n.a.		n.a.
2		8–12		1		n.a.		n.a.		n.a.
3		13–17		n.a.		n.a.		1		n.a.
**Total**				4		18		7		2

n.a., not available

**Table 2:** Distribution of participants by age group, sex, and self-report vs. parent report for the focus (group) interviews

Patients’ characteristics		2–7 years			8–12 years			13–17 years			Total
		male	female		male	female		male	female		
Self-report		n.a.	n.a.		4	2		3	0		9
Parent report		5	2		6	4		7	0		24

**Table 3:** Characteristics of focus (group) participants

		*n* (%)	Mean (SD) / Median (25%-Quartil; 75%-Quartil)	Min / Max
***Information about the child***				
Sex	male	18 (75)		
	female	6 (25)		
Age at data collection (in years)			10 (4) / 10 (7;14)	3 / 17
Siblings		19 (79)		
Siblings with ED		10 (40)		
Severe symptoms (anhidrosis or severe hypohidrosis and oligo- or anodontia)		16 (67)		
De novo mutation		7 (29)		
***Parental information***				
Parental gender	male	4 (18)		
	female	18 (82)		

### Category system

The final category system consists of six main categories, which were initially created deductively from the dimensions of HRQoL: (1) *Physical well-being*, (2) *Emotional well-being*, (3) *Social well-being*, (4) *Autonomy* and (5) *Childcare/school/education*, and then refined inductively from the issues raised in the interviews. In addition, the dimension (6) *Parental well-being* was inductively added.

The intercoder agreement was 71% in the first round, with 20% of the interviews randomly coded by an independent second coder. In the second round, an agreement rate of 88% was achieved. 562 HRQoL statements were identified and assigned to the six main categories and 45 subcategories.

#### Physical well-being

The dimension *Physical well-being* includes statements about the impact of ED-related symptoms on the physical well-being of the patient and the extent to which the child or adolescent suffers from physical discomfort. Hypo-/anhidrosis, i.e., reduced or completely absent sweating ability, and related well-being in the summer or on hot days was mentioned by all children/adolescents and by 20 parents (the mothers of two girls, whose sweating ability is almost normal, rated this topic as less relevant). Pediatric patients of all ages and sexes felt tired and weak on hot days (7 parent reports, 2 self-reports).

*Sometimes I feel so tired all of a sudden. Not just because I have to get up early in the morning*,* but because it’s just getting really hot and I just want to lie down in the cool […].* (girl, age group 8–12 years)

Overheating also caused stomach aches, headaches (1 parent report, age group 13–18 years), and nausea (1 self-report, age group 8–12 years). One boy (parent report) felt *‘burning inside’* when it was too hot. Some parents also reported that their children often had fever, especially in the first few years of life, which made the children restless and stressed (5 parent reports, all age groups). In addition to the main issue of hypo- or anhidrosis, many other aspects were mentioned: high sensitivity to cold (2 parent reports, age group 2–7 years), stressful outdoor temperature fluctuations (2 parent reports, age group 13–17 years). Parents and children described having to deal with a blocked nose (due to nasal congestion and continuous formation of crusts), which affected breathing, speech, and sleep (4 parent reports, age group 2–7 and 13–17 years, and 1 self-report, age group 13–17 years). Three children also had problems with breathlessness (2 parent reports, age groups 2–7 and 8–12 years, and 1 self-report, age group 13–17 years). Some children were susceptible to light and felt it was disturbing or annoying (2 parent reports, age group 2–7 years, and 1 self-report, age group 13–17 years).

*[…] even in very bright sunlight*,* I need a cap and sunglasses. I have really big problems with the sun.* (boy, age group 13–17 years)

On the other hand, one adolescent stated clearly that he had no problems with sunlight.

The main cutaneous symptom mentioned by the children was itching, which some of them found annoying and bothersome (2 self-reports, age groups 8–12 and 13–17 years, and 1 parent report, age group 8–12 years); one boy did not feel affected according to his mother, although he reported itching himself.

Concerning dentures, some children and parents described getting on well with them. Other children refused to wear dentures because they found them unpleasant in their mouths (3 parent reports, age groups 2–7 and 13–17 years).

Two topics were explicitly addressed in each interview: eating and sleeping. When asked how the children coped with eating, most (8 of 9) of them reported that they had no problems with eating and could eat anything they wanted. Several parents said the dentures made eating much easier (4 parent reports, all age groups). 50% of the children (13 out of 24) had at least one dental prosthesis at the time of the interview.

Many children with ED apparently slept poorly and restlessly when it was warm and itching at night also affected their sleep (6 parent reports, all age groups, and 2 self-reports, age groups 8–12 and 13–17 years). Other parents reported that their children slept well, but this was also because of air-conditioning (7 parent reports, all age groups, and 3 self-reports, age group 8–12 years). Parents also described numerous doctor visits and hospital stays, especially in the early years (7 parent reports, all age groups). In two parent reports, descriptions of these visits included child behaviors, such as expressed anxiety or fatigue.

#### Emotional well-being

This dimension includes statements about the impact of ED on the children’s emotional well-being. The reduced ability to sweat also plays an important role here: several children felt *‘unable to be themselves’*,* ‘stuck’ in their bodies*,* irritable*,* and in a bad mood*’ (5 parent reports, all age groups, and 2 self-reports, age groups 2–7 and 13–17).

*When it’s hot*,* and I get*,* well*,* what do you call it*,* a bit… unbearable*,* then I get angry more quickly. And then when [my parents] notice that*,* they say*,* yeah*,* cool down or something like that.* (boy, age group 13–17 years)

Many children described the heat as annoying, disruptive, and stressful (6 self-reports, age groups 8–12 and 13–17 years). Parents’ reports (*n* = 5; all age groups) confirmed this. However, some children did not feel particularly affected by the heat and reported that they had adapted and were used to dealing with it (2 self-reports, age group 13–17 years). Another issue raised exclusively by parents was grooming (skin, nose, etc.), which children found annoying and often refused to do (6 parent reports, all age groups).

Some parents stated that their children were okay with going to the doctor (2 parent reports; age groups 2–7 and 13–17 years), while others said their children were anxious (2 parent reports, age group 2–7 years). Children found dental and orthodontic treatment unpleasant (3 parent reports and 1 self-report, age group 8–12 years). Regarding the children’s life satisfaction and general mood, the statements were predominantly positive: The children appeared to be happy and satisfied despite ED (13 parent reports, all age groups, and 3 self-reports, age groups 8–12 and 13–17 years).

Further aspects of emotional well-being are the children’s self-esteem and self-confidence as well as their satisfaction with their appearance, body, and physical performance. In particular, the younger children (up to the age of 12 years) had a notably positive self-perception and apparent confidence in managing their condition. They were satisfied with their appearance and body, and explicitly also with ED-related features such as thin hair and missing or differently shaped teeth (10 parent reports, age groups 2–7 and 8–12 years, and 2 self-reports, age groups 2–7 and 8–12 years). While this could be interpreted as high self-efficacy, we cannot rule out that it may, in some cases, also reflect a level of limited awareness regarding the long-term implications of their ED.

Many children did not feel different from others or had accepted their peculiarities and did not feel burdened by them (9 parent reports, age groups 2–7 and 8–12 years, and 2 self-reports; age groups 8–12 and 13–17 years). Other children were happy and self-confident overall but had individual things they were not satisfied with because they saw themselves as abnormal in these things and wanted to be like everyone else. Two boys (age groups 8–12 and 13–17 years) described the first time they realized they were different when they experienced restrictions in eating.

*[I also] realized [for the first time that something was different] in kindergarten. We always had a muesli day […]. And I didn’t have that many teeth yet*,* so I couldn’t chew the muesli. And that’s when I realized I was a bit different from the other children.* (boy, age group 13–17 years)

In addition to the restrictions in eating, thin hair and missing teeth were frequently mentioned (6 parent reports, all age groups, and 3 self-reports, age group 13–17 years). Male children and adolescents in particular reported that they felt they were not as physically attractive and fit as others. They described experiences of being a burden and, therefore, being dissatisfied (3 parent reports, age groups 8–12 and 13–17 years).

Finally, this dimension reflects what it is like for the child to have ED and what role ED plays in the child’s perception. For many affected children, ED does not seem to play a significant role in their lives – other things (e.g., relationships with siblings, school grades, toys) appeared to be more critical (13 parent reports, all age groups, and 5 self-reports, age groups 8–12 and 13–17 years).

Some children even mentioned advantages of ED, such as not having to brush their teeth because they do not have them, getting heat-free days at school when it is hot, or being recognized because of their special appearance (3 self-reports, age group 13–17 years).

*Well*,* one advantage is that you can’t sweat*,* […] if you sweat*,* it smells at some point. And that’s why we don’t stink as fast as the others.* (boy, age group 13–17 years)

*I think it’s […] cool to be recognized. […] [My appearance] is a distinctive feature. (boy*,* age group 13*–*17 years)*

Finally, many parents and children reported that it was normal for them to have ED and that they had accepted it (8 parent reports, all age groups, and 3 self-reports, age groups 8–12 and 13–17 years).

In addition to these positive attitudes, negative points of view and thoughts about ED were also reported. For example, one boy (age group 13–17 years) said he usually tried to ignore his condition. A mother reported that her adolescent son did not like to talk about his ED (he did not participate in the interview), and another boy (age group 8–12 years) mentioned that he wishes *‘the illness was out of me.’* One adolescent expressed negative, life-denying thoughts. The description of emotional well-being did not emerge as a dichotomous either–or construct. Rather, in the perceptions of both children and their parents, an ambivalent experiential structure became evident: on the one hand, positive feelings were reported, on the other hand, uncertainties and negative emotions were also expressed. These findings highlight the multidimensionality of emotional experiences, which can coexist simultaneously.

#### Social well-being

The dimension *Social well-being* describes the child’s well-being in the family and among friends and how the subject feels ED is affecting his/her friendships. It represents the perceived acceptance and appreciation by other children, peers, and adults at school, kindergarten, or daycare, and in the leisure time as well as the experience of positive feelings of belonging to a group.

Most children had many good friends and did not feel that ED harmed their friendships. They felt accepted and included (7 parent reports, all age groups, and 5 self-reports, age groups 8–12 and 13–17 years). In addition, some children reported that they received support from their friends, for example, when they struggled with the heat (1 parent report, age group 8–12 years, and 4 self-reports, age groups 8–12 and 13–17 years). On the other hand, two parents stated that their children suffered from being unable to visit friends and participate in recreational activities on hot days (age groups 8–12 and 13–17 years).

However, there are children who have no friends because their peers ostracized them. One mother of a young boy said: *‘At the end of the day*,* he only has his siblings and his big brother’s friends here*,* who are more understanding and also care for him’.* Another boy (age group 13–17 years) described that he had no friends for a long time.

According to many parents, children experienced contact with other affected children as empowering and identity-building (7 parent reports, age groups 8–12 and 13–17 years, and 1 self-report, age group 8–12 years). Some parents also reported that their children would feel a sense of belonging in an institution where other children with disabilities were present because they were *‘not alone in being different’* (3 parent reports, age groups 2–7 and 8–12 years).

Besides social acceptance, a major theme in the interviews was the experience of bullying and exclusion. One boy (age group 13–17 years) described high psychological stress due to severe bullying, which required psychological treatment.

Parents and children also explained that they felt hurt and sad because of mean comments (2 parent reports, age groups 2–7 and 8–12 years and 3 self-reports, age groups 8–12 and 13–17 years). One mother of a boy in puberty reported that her son could handle comments well and didn’t suffer much from them.

Children were challenged by the experience that other children stared at them, looked at them unpleasantly, or excluded them because they looked different or were not as capable (3 parent reports, age groups 2–7 and 8–12 years, and 2 self-reports; age group 13–17 years).

There were only positive statements regarding family well-being and perceived parental support (7 parent reports, all age groups, and 4 self-reports, age groups 8–12 and 13–17 years). Several children (4 out of 9) reported to experience love and appreciation from their families. They also received support for their special needs related to ED, for example, when they felt excluded by peers or restricted by the heat.

#### Autonomy

The dimension *Autonomy* describes statements about the possibilities of doing what the persons wants to do. This includes outdoor (leisure) activities and sports in the summer and the extent to which the child feels restricted and suffers from the restrictions.

On the one hand, some children did not feel significantly restricted in this respect (7 parent reports, all age groups, and 2 self-reports, age group 13–17 years). On the other hand, many children experienced considerable restrictions with regard to outdoor activities, sports, hobbies, or the opportunity to run around on hot days and would become sad, dissatisfied, or frustrated as a result (8 parent reports, all age groups, and 2 self-reports, age group 8–12 years). Children often had to stay indoors on hot days because even short periods outdoors or short distances, such as walking from the parking air-conditioned car to the swimming pool, appeared too long.

*He has a hard time in the heat. For him*,* it starts at 20 or 22 degrees*,* which is too hot for any kind of physical activity. He would really like to join a football club*,* but he knows his own limits and that’s something he really struggles with.* (parents of a boy, age group 8–12 years)

However, having a pool in the garden or being close to some form of water (public swimming pool, lake, etc.) gives the children the opportunity to be outside even in warm weather (4 parent reports, age groups 2–7 and 8–12 years, and 3 self-reports, age group 8–12 years).

#### Child care/school/education

The dimension *Child care/school/education* focuses on the children’s well-being at school, kindergarten, or daycare. In addition to statements about acceptance by classmates (which are discussed under the dimension *Social well-being*), the main topic related to school that was addressed were again the reduced or lacking ability to sweat and the resulting lack of well-being in the summer.

One girl (age group 8–12 years) described her school experience as exhausting in the heat. Some children reported that the classroom was too warm during the summer and that they felt more distracted (1 parent report, age group 13–17 years, and 2 self-reports, age groups 8–12 and 13–17 years). Another issue was school sports, in which some children were unable to participate in the summer months (2 parent reports and 3 self-reports; age group 8–12 years). However, some of these children described this as ‘okay’ and mentioned that they did not find it annoying.

A mother also reported that her son (age group 13–17 years) could not go to school at all when the air temperature was too high. Most children experienced support and understanding from their teachers regarding their reduced heat tolerance.

#### Parental well-being

The dimension *Parental well-being* includes statements about how the parents feel when they have a child affected by ED, their joys and worries and challenges in daily life.

On the one hand, all the parents reported that they enjoyed their children and had gained a new perspective on life and what is essential in life. *‘[…] he is just so extremely happy and has totally enriched us and is funny.’* (parent of a boy, age group 8–12 years). Many mothers and fathers, including those of younger children (2–7 years), also stated that ED was well integrated into their everyday lives and did not play a major role (14 parent reports, all age groups).

On the other hand, parents described worries, anxiety, and stress. Those who had younger sons (2–7 years) with anhidrosis worried that their children would not react correctly if they overheated and that they would be unable to help themselves when overheating without their supervision. A second concern mentioned was that their child could choke while eating.

Ten parents also described the fear of their child being bullied, three were concerned about their children’s future, for example, their future careers. Another aspect relates to ED-specific measures, such as special skin, eye, nose or ear care and the wearing of dentures. These measures were often refused by younger children and by adolescents, which caused stress to the caregiver.

In addition, some parents had experienced challenges in dealing with health and care insurance companies. The German social security systems offer financial and practical support to individuals requiring long-term care due to disability or chronic illness. This support may include coverage of costs for dental prostheses or air conditioning systems (3 parent reports, age group 2–7 years). Only a few felt well-supported when dealing with doctors (2 parent reports, age group 13–17 years). The majority thought they were not taken seriously, especially in emergencies, and doctors were uninformed about the condition (8 parent reports; age groups 2–7 and 13–17 years).

### Item development

Based on the 562 HrQoL statements of children and adolescents with ED aged 2 to 17 years and their parents, we built an item pool for the pilot test version of the ED-QoL (Table 4). Particular attention was paid to keeping the wording as closely as possible to the original statements. From the cognitive debriefing part of the focus group interviews, 164 statements related to the second preliminary, non-validated questionnaire were collected, covering content, time frame, response categories, comprehensibility, practicability, and difficulties in completion which we considered when creating the pilot-ED-QoL.

**Table 4:** Examples of item development according to main dimensions

Dimension		Example statement from the interviews		Pilot test item
Physical well-being		*‘Yes*,* I’ve noticed that I’m getting too warm and that I’m starting to feel weak*,* exhausted. I think I’m at the point where I can’t do it anymore.’* (boy, age group 8–12 years)		I felt exhausted.
		*‘He says he feels like he’s on fire*,* like everything inside him is burning. I can’t really picture what that’s like. I don’t know what it’s like*,* but he’s really struggling then.’* (parents of a boy, age group 13–17 years)		I felt like I was burning inside.
		*‘The only thing that seems a bit critical to me now is the light. I can’t leave the house without sunglasses in summer anymore. […] I need a cap and sunglasses*,* even in very light sunlight. I really have big problems with the sun.’* (boy, age group 13–17 years)		I had problems with my eyes because the light was blinding.
Emotional well-being		*‘So when it’s hot for me and I get*,* yes*,* what’s the word*,* a bit… unbearable*,* so I get annoyed more quickly*,* so. And then when they [my parents] notice that*,* they say*,* yes*,* cool down or something. […] I usually don’t want to accept that.’* (boy, age group 13–17 years)		I was easily irritable.
		*‘I’m happy with my body in all its forms. I even like my teeth. Teeth are the biggest issue for me […]’* (girl, age group 8–12 years)		I felt satisfied with my body.
		*‘I don’t have to brush my teeth. That’s one of the advantages of being me. I save on toothpaste and toothbrushes*,* which is a bonus.* (boy, age group 13–17 years)		I saw benefits in my ED.
Social well-being		*‘But otherwise [except for the one question of whether what I have is contagious]*,* everyone has just reacted normally*,* as if it were nothing special*,* and everyone treats me the same as they treat everyone else.’* (girl, age group 8–12 years)		I was treated like everyone else.
		*‘When we go to the playground in our neighbourhood […] other children just stare at him and then walk away. Some are afraid*,* yes. Some of them even laugh at him.’* (parents of a boy, age group 2–7 years)		I was excluded because of my ED.
		*‘I did okay at primary school*,* but it was only at secondary school that I really struggled. I was bullied and teased*,* and I still get teased to some extent today.’* (boy, age group 13–17 years)		I was bullied because of my ED.
Childcare/ School/ Education		*‘I get on really well with school in general*,* apart from the ED. My teacher is great at dealing with it.’* (girl, age group 8–12 years)		The teachers supported me with my ED.
		*‘He’s not at school at the moment because it’s too warm. It’s currently 30 degrees here. He doesn’t have any sweat glands. It’s not feasible to go there and back and the school building is far too warm.’* (parents of a boy, age group 13–17 years)		I missed school because of my ED.
Parental well-being		*‘The main issue at the moment is actually swallowing while eating. […] We’ve already had two incidents where something has really got stuck. […] I’m afraid that when I’m alone with him*,* I sometimes have to sweat blood and water just to make sure everything goes well.’* (parents of a boy, age group 2–7 years)		I was worried that my child would choke while eating.
		*‘The biggest issue I saw was that she might be bullied because of her appearance. Fortunately*,* the children in kindergarten accepted her for who she was. […] And now*,* two years ago*,* when she changed schools*,* I was worried again that she would be bullied.’* (parents of a girl, age group 8–12 years)		I was afraid my child would be bullied.
		*‘Of course*,* I have big worries about what it will be like when he’s older. What if he falls in love with a girl and she realizes he only has dentures and no real teeth? I’m also worried about what it will be like for him in his professional life.’* (parents of a boy, age group 2–7 years)		I was worried about my child’s future.

The pilot questionnaire included four core dimensions: *Physical well-being* (24 items), *Emotional well-being* (22 items), *Social well-being* (15 items) and *Childcare/School/Education* (5 items). Additionally, the dimension *Parental well-being* (17 items) was integrated. Items related to the dimension *Autonomy* were assigned to the two core dimensions *Social well-being* and *Emotional well-being*.

The pilot ED-QoL was developed in two age-adjusted versions: (1) for parents of children aged 2–7 years as an observer report, and (2) for children and adolescents aged 8–17 years both as a self-report form and an observer report, while the items for self-report correspond exactly to the proxy items but are presented from a first-person perspective (Table 5).

**Table 5:** List of items for the pilot test version of the questionnaire (proxy reports, 2–17 years)*

**Physical domain (24 items)**	**Emotional domain (22 items)**	**Social domain (15 items)**
My child felt exhausted.	My child was annoyed by not being able to sweat.	My child felt supported by his/her family with the ED.
My child felt uncomfortably hot.	My child was easily irritable.	My child felt understood by his/her family with the ED.
My child felt uncomfortably cold.	My child felt like he/she was no longer himself.	My child was supported by his/her friends with the ED.
My child had a headache.	My child felt like he/she was stuck in his/her body.	My child’s friends adapted to his/her needs (e.g. playing in the shade/inside).
My child had a stomachache/nausea.	My child’s family supported him with the ED.	My child felt treated like everyone else.
My child felt like he/she was burning inside.	My child knew what to do when he/she was too hot.	Others accepted my child as he/she is.
My child had skin problems (e.g., dry, chapped, sore).	My child was sad because he/she couldn’t do what he/she wanted because of the ED.	My child was excluded because of his/her ED.
My child had itching of the body.	My child was angry because he/she couldn’t do what he/she wanted because of the ED.	My child was bullied because of his/her ED.
My child had breathing problems.	My child was satisfied with the way he/she looked.	Others stared at my child because of his/her ED.
My child had a stuffy nose.	My child felt satisfied with his/her body.	My child was able to talk openly about his/her ED with others.
My child had severe throat irritation (caused by nasal mucus).	My child felt that he/she was different.	Not being able to sweat has restricted my child in his/her daily life.
My child had dry mouth problems.	My child had psychological problems (e.g. outbursts of anger, anxiety) because of the ED.	My child was restricted in his/her leisure activities (e.g. sports) because of the ED.
My child had problems with his/her eyes because the light was blinding.	My child was annoyed by his/her ED.	Visiting the doctor has restricted my child’s free time.
My child had eye problems because they itched/watered.	My child was disturbed by the care measures (e.g. having his/her nose sucked out, applying cream).	My child’s ED determined where he/she stayed.
My child had hearing problems (due to blocked ears).	My child didn’t like taking eye drops.	My child was able to do what others did.
My child’s voice bothered him/her (scratchy, high-pitched, rough).	My child didn’t like going to the doctor (e.g. dentist).	
Other people had trouble understanding my child when he/she spoke.	My child felt uncomfortable when someone asked about his/her ED.	**Educational domain (5 items)**
My child was able to eat everything he/she wanted to eat.	My child thought it was unfair that he/she had ED and others did not.	My child was unable to pay attention well at kindergarten/school/vocational training.
My child felt able to perform.	My child accepted his/her ED.	My child’s special needs were taken into account in kindergarten/at school/during training.
My child was able to sleep well at night.	My child saw benefits in his/her ED.	My child’s teachers supported him/her with the ED.
My child wet the bed at night.	My child knew who to turn to when he/she was not feeling well (e.g., when he/she was sad).	My child missed kindergarten/school/training because of his/her ED.
My child had to be treated in hospital for an infection.	My child was confident.	My child was bothered by having to take breaks from playing/sports at kindergarten/school/training.
My child found it difficult to wear dentures.		
My child experienced pain due to dentures.		
**Effects on parents (17 items)**		
I knew what I could do if my child overheated.	It was stressful for me that I had to force my child to take ED-specific measures (e.g. care measures, wearing the prosthesis).	I was worried about my child’s future.
I was worried that I wouldn’t react properly if my child overheated.	It was stressful for me to have to educate others about my child’s ED.	I like my child the way he/she is.
I was worried that my child would not be able to help themselves if they overheated.	I knew who I could turn to if I needed support.	I felt well supported by the doctors treating my child.
I was worried that my child would choke while eating.	I was afraid my child would be bullied.	I did not feel taken seriously by the doctors treating my child in emergency care.
I found preparing the food (e.g. cutting it into small pieces) time-consuming.	I was overprotective.	I experienced contact with the statutory health/ long-term care insurance fund as stressful.
It was stressful for me that my child took a long time to eat.	I was emotionally distressed that my child had ED.	

* The items for self-report correspond exactly to the proxy items. The only difference is that they are presented from a first-person perspective

## Discussion

This study is the first that examined the HRQoL of children and adolescents with hypohidrotic ED through qualitative interviews. It provides comprehensive insight into the feelings and subjective experiences of affected children and adolescents.

To the best of our knowledge, there is only one study on the quality of life of children and adolescents with XLHED using generic questionnaires [8], which showed hypohidrosis to be a significant factor for low quality of life.

Our results confirm that repeated hyperthermia as a consequence of absent or deficient sweat glands has the potential to negatively impact the quality of life in the summer months, affecting all its dimensions. With regard to the physical well-being, it can be impaired due to various symptoms: from weakness and fatigue to abdominal discomfort, nausea, and headaches. Notably, a boy described a feeling of *‘burning inside’*, illustrating the severity of the issue. Perceived as bothersome and distressing, it can also impact the emotional well-being, leading to irritability, depressed mood, and the sensation of no longer feeling themselves because of being trapped in their overheating bodies. Such statements highlight mental suffering due to physical restrictions. Some study participants, especially male children and adolescents, perceived the limited physical capacity to be emotionally distressing, as they felt not as fit as others. This observation aligns with existing literature, which links physical limitations with deleterious effects on self-esteem. Specifically, Pinquart emphasized the particularly harmful influence of physical limitations on self-esteem among male adolescents [21]. Concerning autonomy and social challenges, young patients reported that they were sad, dissatisfied, or frustrated due to restrictions on outdoor, sports, and leisure activities during the summer. Affected children had to stay indoors on sunny days, impairing their ability to socialize. Our study also shows that children with ED might experience high levels of stress during the school day, particularly in a warm environment. One mother reported that her son was frequently unable to attend school during the summer months. As participation in school activities is important for developing self-esteem, frequent absence due to chronic illness leads to academic failure and self-esteem challenges [22]. Teacher support has a significant effect on the well-being of affected children, as observed in studies on children and adolescents with other rare diseases [23]. Professional insights align with the findings [24]. In our study, most children experienced support from their teachers with respect to the reduced heat tolerance.

In these detailed descriptions of sensations associated with overheating, our study provides a deeper understanding of the individual experiences of patients than a previous study which used only dermatologic QoL questionnaires suggesting that “school/work activities, personal relationships, and social functioning did not seem to be affected by ED” [8]. Such statements broaden our current discourse and offer starting points for improving care and quality of life.

In addition to hyperthermia, other aspects were also reported in the physical well-being dimension, such as sensitivity to light and itching, which were perceived as bothersome. Itching, a stuffed nose, and overheating resulted in sleep disturbances. Frequent medical visits and hospitalizations caused stress to families. These findings highlight new issues that previous studies didn’t address [8] and provide clear recommendations, such as the use of air conditioning in bedrooms and consistent skin and nose care.

With regard to emotional well-being, children experienced “being different” as a concern, a phenomenon also documented for other chronic illnesses [21]. The additional influence of appearance on self-image is well documented [25, 26]. Some study participants reported strong feelings of dissatisfaction due to physical imperfections. These observations point to the effectiveness of reconstructive procedures in improving quality of life [27]. Research further indicates that stigmatization and a negative body image are highly prevalent among individuals with chronic diseases, particularly among adolescents. Pinquart posits that disease-related limitations can clearly impact self-esteem and mental health [21]. Nevertheless, most children reported positive emotions with regard to life satisfaction and acceptance of their condition, particularly in the younger group, similar to findings in other studies on chronic pediatric diseases [28, 29]. Some even described benefits, such as fewer dental routines and school days, and unique attention due to their characteristics. Positive perceptions indicate potential coping strategies [30]. A finding of our study was the self-reported high level of confidence among a subset of patients despite the challenges associated with ED. This observation invites a nuanced interpretation. While it likely reflects genuine psychological resilience and successful adaptation to a lifelong condition, the role of illness perception must be considered. The large clinical variability of ED means that individuals with non-syndromic or milder phenotypes might perceive their condition as less impactful [31]. Furthermore, this self-assessment may vary significantly with age, as the primary concerns and psychosocial challenges of ED evolve from childhood through adulthood, influencing self-concept differently across the lifespan.

Concerning the social well-being, a major theme was the exposure to bullying and exclusion. Some children described a lack of support from peers, leading to sadness and pain. Studies have shown that stigma in pediatric patients with chronic skin diseases is linked to a lower quality of life [32]. Beyond this negative experience, most children with ED and their parents reported that the children feel accepted, maintain friendships and receive love and support from their parents. Trusting in the support of parents and friends is crucial to the mental well-being of children and young adults who cope with chronic illnesses. Reliable relationships give emotional security and enhanced coping mechanisms. Parents play a pivotal role by providing care and advocacy. They also create a nurturing environment characterized by understanding and stability. Friendships are also important sources of support, belonging, and normalcy, helping prevent social isolation, which is especially relevant for children and adolescents with visible or limiting illnesses. Social integration is the key to reducing stigmatization and distress [33]. Contact with other ED patients was perceived as positive, with meetings in patient organizations being particularly helpful. This agrees well with the observations of Delisle [34], who emphasized the identity-building and empowerment that patient support groups offer to their members. Meetings provide a platform for sharing experiences and community development.

Furthermore, the interviews conducted in this study provide valuable information about the well-being of parents, which has been shown to impact parents perception of their children’s quality of life [35]. A specific section was created in the parent questionnaire to assess parental well-being and identify potential needs for support. Such information could help medical professionals recommend targeted psychological support when necessary. Many parents reported that their children’s illness had given them a new perspective on life. However, they also expressed concerns about the caregiving responsibilities for a child with ED, including fears about overheating and choking during meal times. Contact with health insurers and other institutions was also perceived as very stressful. Some parents additionally stated that medical professionals were not always adequately informed about ED. Thus, our research may help to improve physicians’ level of information and thereby improve the parental quality of life in the context of healthcare facilities.

Many findings of this study are consistent with the results of Dufrense et al. who developed an ED-specific burden of disease score based on parent interviews [36], covering issues such as fears or concerns about the child’s future, impairment of the parents’ social life due to eating difficulties, helplessness in the face of the child’s symptoms, difficulties in persuading the child to accept dental care, and the suffering of seeing the child being teased because of his/her illness (cf. Table 5, effects on parents). The reported stressors are similar to those stated by parents of children with other chronic conditions, such as concerns about bullying, worries regarding their children’s future careers, and general uncertainty about their future development [37]. Nevertheless, this additional scale on parental well-being is not intended to provide a comprehensive assessment of parents’ quality of life or burden of disease, but rather to complement the patient perspective by capturing the family perspective and the broader impact of ED on the family system.

The results of our study also demonstrate that the QoL of children and adolescents with ED is multifaceted and can vary. Consequently, positive and negative experiences were coded in all dimensions. This underscores the notion that children and adolescents with chronic illnesses cannot be presumed to have a profoundly diminished sense of well-being [21].

### Practical implications

The item development for a condition-specific PROM offered insights into the challenges and positive aspects of living with ED. Results indicate that potential coping strategies can lead to acceptance and a satisfactory quality of life. It is imperative to provide adequate support options for those who perceive ED mainly as a burden, with the aim of enhancing their well-being and facilitating more active participation in life.

Our findings highlight the importance of peer networks and support group meetings for children and adolescents with this rare condition. Sharing experiences can boost self-esteem, reduce social isolation, and improve quality of life. Inclusive, supportive educational environments are also crucial. Educators must understand and meet the needs of children with ED, including adequate room temperatures, individualized support, and offering alternatives to certain sports activities. These measures can reduce stress, promote integration, and enhance the well-being of affected children in school. The data underscore the necessity of a comprehensive approach that also addresses the needs of parents. Support services can alleviate parental stress by reducing the burdens faced by families and improving QoL for children and their caregivers.

The novel insights into the HRQoL experiences of children and adolescents with ED emphasize the perceived influence of heat intolerance on daily life, the emotional burden of physical limitations, and the importance of coping strategies, social inclusion, and supportive relationships. The results thus do not only contribute to a more profound understanding of patients’ experiences, but may also guide ongoing clinical research [38–40] and current practice.

This study represents a crucial first step in the development of a disease-specific HRQoL instrument for HED by generating a comprehensive, patient-centered item pool based on qualitative data. The next essential phase will be to administer these items in a larger sample to empirically examine the scale’s psychometric properties, including its factor structure, reliability, and validity. Therefore, our work should be seen as a foundational exploratory study that enables and informs future quantitative validation. The stepwise approach is consistent with best practices in PROM development (FDA guidelines).

### Strengths and limitations

Qualitative interviews were conducted with both children and their parents, which improves data collection and allows to gain broader and more meaningful insights into what ED means to the whole family system. However, the study is subject to several limitations: The conclusions are based on a small sample, due to the rarity of the disease, which severely limits the generalizability of the results. In addition, the study was monocentric, which means that possible regional or institutional differences could not be considered.

Our sample consisted primarily of male adolescents; thus, the perspective of affected female adolescents may be missing. The predominance of male adolescents may have led to an emphasis on certain types of psychosocial burdens, and future research should actively seek to include female adolescents with ED to provide a more comprehensive view.

A selective participation of families with greater challenges or burdens would be particularly problematic. The sample’s selective characteristics, such as language, cultural background, and a tendency for participants to have a higher socioeconomic status, further limit the generalizability of the results. Furthermore, while this study collected both self- and proxy reports to gain a comprehensive understanding of HRQoL, the qualitative design did not allow for a quantitative dyadic analysis of the interdependence between child and parent well-being. This mutual influence is a known methodological challenge in pediatric PROM development, and future quantitative phases of research will specifically examine such parent-child dyads to disentangle overlapping perspectives.

Although our qualitative approach included perspectives from both mothers and fathers, the design did not allow for a systematic examination of potential gender differences in caregiving roles and psychological strain. This important aspect will also be a focus of subsequent research.

The sample contains a higher proportion of parent reports than adolescent self-reports due to differential recruitment and response rates. While these perspectives are considered complementary, the imbalance is noted. Although children and adolescents were instructed to participate in a private setting, the remote nature of the online interviews means that we cannot guarantee that parents were not present. This introduces a potential, unquantifiable risk of social desirability bias, particularly for sensitive topics, which should be taken into account when interpreting the findings.

We offered participants a choice between focus groups and individual interviews to accommodate preferences and maximize comfort. While this approach enriched our data by capturing both shared and highly individual perspectives, the inherent characteristics of each method must be acknowledged. Data from focus groups may reflect group dynamics and consensus, whereas individual interviews provide deeper, personal narratives. This methodological blend is a key consideration when interpreting the spectrum of experiences presented.

## Data Availability

The datasets used and analyzed in this study are available from the corresponding author upon reasonable request.

## References

[1] Peschel N, Wright JT, Koster MI, et al. Molecular pathway-based classification of ectodermal dysplasias: first five-yearly update. Genes. 2022;13(12):2327. 36553593 10.3390/genes13122327 PMC9778228

[2] Herlin LK, Schmidt SAJ, Hermann XB, et al. Prevalence and patient characteristics of ectodermal dysplasias in Denmark. JAMA Dermatol. 2024;160(5):502–10. 38477886 10.1001/jamadermatol.2024.0036 PMC10938247

[3] Bullinger M. Das Konzept der Lebensqualität in der Medizin – Entwicklung und heutiger Stellenwert. Z Evid Fortbild Qual Gesundhwes. 2014;108(2–3):97–103. 24780706 10.1016/j.zefq.2014.02.006

[4] Felder-Puig R, Topf R, Maderthaner R, Gadner H, Formann AK. Konzept der „gesundheitsbezogenen Lebensqualität in der Pädiatrie. Monatsschr Kinderheilkd. 2009;157(7):675–82.

[5] Ravens-Sieberer U, Voss C, Reiss F, Wüstner A, Otto C. Messung gesundheitsbezogener Lebensqualität Im Kindes- und jugendalter. Public Health Forum. 2019;27(3):177–82.

[6] Calvert MJ, Cruz Rivera S, Retzer A, et al. Patient-reported outcome assessment must be inclusive and equitable. Nat Med. 2022;28(6):1120–4. 35513530 10.1038/s41591-022-01781-8

[7] Clarke A, Phillips DI, Brown R, Harper PS. Clinical aspects of X-linked hypohidrotic ectodermal dysplasia. Arch Dis Child. 1987;62(10):989–96. 2445301 10.1136/adc.62.10.989 PMC1778691

[8] Pavlis MB, Rice ZP, Veledar E, Bradley BR, Spraker MK, Chen SC. Quality of life of cutaneous disease in the ectodermal dysplasias. Pediatr Dermatol. 2010;27(3):260–65. 20609143 10.1111/j.1525-1470.2010.01121.x

[9] Guntreddi G, Vasudevan Nair J, Nirujogi SP. Ectodermal dysplasia presenting as heat exhaustion in an adolescent Boy. Cureus. 2021;13(2):e13450. 33767934 10.7759/cureus.13450 PMC7983733

[10] Hammersen JE, Neukam V, Nüsken KD, Schneider H. Systematic evaluation of exertional hyperthermia in children and adolescents with hypohidrotic ectodermal dysplasia: an observational study. Pediatr Res. 2011;70(3):297–301. 21646941 10.1203/PDR.0b013e318227503b

[11] Hummel P, Guddack S. Psychosocial stress and adaptive functioning in children and adolescents suffering from hypohidrotic ectodermal dysplasia. Pediatr Dermatol. 1997;14(3):180–85. 9192408 10.1111/j.1525-1470.1997.tb00233.x

[12] Blumer S, Bogachek-Halfon L, Peretz B, Shpack N, Nissan S. Parental perceptions of prosthetic treatment for and coping abilities of children with ectodermal dysplasia: a pilot study. Pediatr Dent. 2018;40(7):449–52. 31840646

[13] Crossan E, O’Connell AC. Parental perception on oral health-related quality of life and dental features of ectodermal dysplasia and isolated hypodontia in children. BMC Oral Health. 2021;21(1):510. 34627220 10.1186/s12903-021-01878-5 PMC8502392

[14] Niekamp N, Kleinheinz J, Reissmann DR, Bohner L, Hanisch M. Subjective oral health-related quality of life and objective oral health in people with ectodermal dysplasia. Int J Environ Res Public Health. 2020;18(1):1–10. 33379169 10.3390/ijerph18010143 PMC7796382

[15] Benjamin K, Vernon MK, Patrick DL, Perfetto E, Nestler-Parr S, Burke L. Patient-reported outcome and observer-reported outcome assessment in rare disease clinical trials: an ISPOR COA emerging good practices task force report. Value Health. 2017;20(7):838–55. 28712612 10.1016/j.jval.2017.05.015

[16] US Department of Health and Human Services Food and Drug Administration. Guidance for industry: patient-reported outcome measures: use in medical product development to support labeling claims [Internet]. 2009 [cited 2024 June 20]. Available under https://www.fda.gov/media/77832/download.

[17] Kuckartz U, Rädiker S. Fokussierte Interviewanalyse mit MAXQDA: Schritt für Schritt. 2. Auflage. Wiesbaden, Heidelberg: Springer VS. 2024.

[18] Kuckartz U. Qualitative Inhaltsanalyse. Methoden, Praxis, Computerunterstützung. 4., überarbeitete Aufl. Weinheim: Beltz. 2018.

[19] Schilb S. Card Sorting – Techniken Im Überblick. i-com. 2005;4(1):49–50.

[20] Wild D, Grove A, Martin M, et al. Principles of good practice for the translation and cultural adaptation process for Patient-Reported outcomes (PRO) measures: report of the ISPOR task force for translation and cultural adaptation. Value Health. 2005;8(2):94–104. 15804318 10.1111/j.1524-4733.2005.04054.x

[21] Pinquart M. Wenn kinder und Jugendliche körperlich Chronisch Krank Sind. Berlin, Heidelberg: Springer; 2013. pp. 36–50.

[22] Pinquart M, Teubert D. Academic, physical, and social functioning of children and adolescents with chronic physical illness: a meta-analysis. J Pediatr Psychol. 2012;37(4):376–89. 22173882 10.1093/jpepsy/jsr106

[23] Witt S, Schuett K, Wiegand-Grefe S, Boettcher J, Quitmann J. Living with a rare disease – experiences and needs in pediatric patients and their parents. Orphanet J Rare Dis. 2023;18(1):242. 37568186 10.1186/s13023-023-02837-9 PMC10422846

[24] Witt S, Kristensen K, Wiegand-Grefe S, et al. Rare pediatric diseases and pathways to psychosocial care: a qualitative interview study with professional experts working with affected families in Germany. Orphanet J Rare Dis. 2021;16(1):497. 34838091 10.1186/s13023-021-02127-2 PMC8626925

[25] Pinquart M. Body image of children and adolescents with chronic illness: a meta-analytic comparison with healthy peers. Body Image. 2013;10(2):141–8. 23219705 10.1016/j.bodyim.2012.10.008

[26] Vivar KL, Kruse L. The impact of pediatric skin disease on self-esteem. Int J Womens Dermatol. 2017;4(1):27–31. 29872673 10.1016/j.ijwd.2017.11.002 PMC5986112

[27] Deo K, Sharma YK, Shah B, et al. Improvement in the quality of life of a patient of ectodermal dysplasia with reconstructive surgeries. J Cutan Aesthet Surg. 2019;12(4):244–47. 32001971 10.4103/JCAS.JCAS_17_19 PMC6967165

[28] Blackwell CK, Elliott AJ, Ganiban J, et al. General health and life satisfaction in children with chronic illness. Pediatrics. 2019;143(6):e20182988. 31061222 10.1542/peds.2018-2988 PMC6564050

[29] Kovacs KE, Boris P, Nagy BE. Psychological well-being and life satisfaction in children and adolescents with chronic illness: the role of depression, nonproductive thoughts, and problematic internet use. Children. 2025;12(5):657. 40426836 10.3390/children12050657 PMC12109936

[30] Compas BE, Jaser SS, Dunn MJ, Rodriguez EM. Coping with chronic illness in childhood and adolescence. Annu Rev Clin Psychol. 2012;8:455–80. 22224836 10.1146/annurev-clinpsy-032511-143108 PMC3319320

[31] Burger K, Schneider AT, Wohlfart S, et al. Genotype-phenotype correlation in boys with X-linked hypohidrotic ectodermal dysplasia. Am J Med Genet Part A. 2014;164:2424–32. 10.1002/ajmg.a.36541 24715423

[32] Paller AS, Rangel SM, Chamlin SL, et al. Stigmatization and mental health impact of chronic pediatric skin disorders. JAMA Dermatol. 2024;160(6):621–30. 38656377 10.1001/jamadermatol.2024.0594 PMC11044010

[33] Berntsson L, Berg M, Brydolf M, Hellström A-L. Adolescents’ experiences of well-being when living with a long-term illness or disability. Scand J Caring Sci. 2007;21(4):419–25. 18036004 10.1111/j.1471-6712.2006.00490.x

[34] Delisle VC, Gumuchian ST, Rice DB, et al. Perceived benefits and factors that influence the ability to Establish and maintain patient support groups in rare diseases: a scoping review. Patient. 2017;10(3):283–93. 28004275 10.1007/s40271-016-0213-9

[35] Witt S, Kolb B, Bloemeke J, Mohnike K, Bullinger M, Quitmann J. Quality of life of children with achondroplasia and their parents – a German cross-sectional study. Orphanet J Rare Dis. 2019;14(1):194. 31399110 10.1186/s13023-019-1171-9 PMC6688231

[36] Dufresne H, Maincent O, Taieb C, Bodemer C, Hadj-Rabia S. The ectodermal Dysplasias-Burden of disease score: development and validation of an ectodermal dysplasia family/parental burden score. Acta Derm Venereol. 2023;103:5203. 37646348 10.2340/actadv.v103.5203 PMC10547059

[37] Witt S, Rohenkohl A, Bullinger M, et al. Understanding, assessing and improving health-related quality of life of young people with achondroplasia – a collaboration between a patient organization and academic medicine. Pediatr Endocrinol Rev. 2017;15(Suppl 1):109–18. 29292874 10.17458/per.vol15.2017.wrm.improvinghealthrelatedquality

[38] Schneider H. Ectodermal dysplasias: new perspectives on the treatment of so Far immedicable genetic disorders. Front Genet. 2022;13:1000744. 36147498 10.3389/fgene.2022.1000744 PMC9485875

[39] Schneider H, Hadj-Rabia S, Faschingbauer F, et al. Protocol for the phase 2 EDELIFE trial investigating the efficacy and safety of intra-amniotic ER004 administration to male subjects with X-linked hypohidrotic ectodermal dysplasia. Genes. 2023;14:153. 36672894 10.3390/genes14010153 PMC9858920

[40] Schneider H, Schneider M, Lia M, et al. Attitudes of female carriers of X-linked hypohidrotic ectodermal dysplasia towards prenatal treatment and their decisions during a pregnancy with a male fetus. Orphanet J Rare Dis. 2025;920:182. 10.1186/s13023-025-03710-7 PMC12001694 40234959

